# Integrated ecdysone and *O-*linked *N*-acetylglucosamine signaling coordinates intestinal stem cell proliferation in *Drosophila* midgut

**DOI:** 10.1093/g3journal/jkaf190

**Published:** 2025-08-19

**Authors:** Hyun-Jin Na, YiSeul Kim, Jong Min Kim, Mi Jeong Sung, Joung-Sun Park

**Affiliations:** Aging Research Group, Food Functionality Research, Korea Food Research Institute, 245 Nongsaenmyeong-ro, Iseo-myeon, Wanju-gun, Jeollabuk-do 55365, Republic of Korea; Aging Research Group, Food Functionality Research, Korea Food Research Institute, 245 Nongsaenmyeong-ro, Iseo-myeon, Wanju-gun, Jeollabuk-do 55365, Republic of Korea; Aging Research Group, Food Functionality Research, Korea Food Research Institute, 245 Nongsaenmyeong-ro, Iseo-myeon, Wanju-gun, Jeollabuk-do 55365, Republic of Korea; Aging Research Group, Food Functionality Research, Korea Food Research Institute, 245 Nongsaenmyeong-ro, Iseo-myeon, Wanju-gun, Jeollabuk-do 55365, Republic of Korea; Institute of Nanobio Convergence, Pusan National University, Busan 46241, Republic of Korea; Department of Molecular Biology, Pusan National University, Busan 46241, Republic of Korea

**Keywords:** *Drosophila*, intestinal stem cell, ecdysone, *O*-GlcNAc, DNA damage response

## Abstract

Steroid hormones and nutrient-sensitive signaling pathways play critical roles in the regulation of stem cell activity, maintenance of tissue homeostasis, and the coordination of metabolic functions. In *Drosophila*, the steroid hormone ecdysone and the nutrient-responsive posttranslational modification *O-*linked *N*-acetylglucosaminylation (*O*-GlcNAcylation) are emerging as key regulators of intestinal stem cell (ISC) behavior. This study aimed to investigate how the interplay between ecdysone signaling and *O*-GlcNAcylation controls ISC proliferation and gut homeostasis, particularly in the context of aging. We showed that ecdysone receptor (EcR) expression increases during aging and upon increased O-GlcNAcylation and that both genetic overexpression of EcR and exogenous 20-hydroxyecdysone treatment promote ISC proliferation and increase *O-*GlcNAc levels. Conversely, the knockdown of EcR or *O-*GlcNAc transferase suppressed ISC proliferation and reduced DNA damage accumulation. Our results show that EcR signaling induces DNA damage response and cooperates with *O-*GlcNAcylation to regulate ISC activity, suggesting a positive feedback loop involving hormones and nutrients. These results highlight the interaction between EcR and *O*-GlcNAc as a metabolic gatekeeper that balances regenerative activity and genomic integrity in the aging gut. These findings provide a potential mechanistic link for therapeutic strategies for age-related and metabolic diseases involving abnormal stem cell proliferation.

## Introduction

Hormones play a fundamental role in the regulation of physiological activity, muscle strength, and body composition (Chikani and Ho 2014). Steroid hormones, such as estrogen, progesterone, and testosterone, serve as key regulators of human physiological function and activity (Chikani and Ho 2014). Steroid hormones have a significant effect on glucose-dependent metabolism, energy balance, and cellular homeostasis and are therefore highly relevant to survival and fitness (Ahmed et al. 2020). Steroid sex hormones are important modulators of nutrient balance and energy metabolism (Mahboobifard et al. 2022). Ecdysone (20-hydroxyecdysone, 20HE) is a steroid hormone synthesized from cholesterol by cytochrome P450 enzyme (Tsukagoshi et al. 2016). Ecdysone regulates the timing and progression of tissue development while affecting the nervous system, reproductive functions, metabolism, and lifespan (Ahmed et al. 2020; Karanja et al. 2022; Wani et al. 2023). In addition, 20HE promoted intestinal stem cell (ISC) proliferation and increased gut size in a female *Drosophila* model system (Ahmed et al. 2020). Despite these established roles, the precise mechanisms, through which ecdysone regulates nutrient-dependent metabolic processes in stem cells, remain poorly understood.

Nutrient-sensitive *O-*linked *N*-acetylglucosamine (*O-*GlcNAc)-ylation, a dynamic posttranslational modification, is markedly elevated in individuals with metabolic disorders and malignancies (Olivier-Van Stichelen et al. 2017; Konzman et al. 2020). This modification regulates a broad range of cellular processes, including the maintenance of pluripotency, embryonic development, intracellular signaling, and interactions with the cellular microenvironment (Jang et al. 2012; Na et al. 2020). Ultimately, *O-*GlcNAcylation influences critical physiological pathways, such as protein folding and metabolic signaling (Konzman et al. 2020). Dysregulated *O-*GlcNAcylation is implicated in the pathogenesis of insulin resistance, obesity, and Alzheimer's disease, highlighting its potential as a therapeutic target for metabolic and age-related disorders (Lockridge and Hanover 2022). In aging organisms, elevated *O-*GlcNAcylation is also associated with increased stem cell activity and disrupted tissue homeostasis (Na et al. 2020). Concurrently, DNA damage response (DDR) functions as a genomic surveillance system that preserves the genomic integrity of proliferative cells, including ISCs (Park et al. 2012; Park et al. 2018). DNA damage accumulation due to aging or oxidative stress activates DDR signaling pathways, such as the ATM/ATR-mediated phosphorylation cascade, which in turn influences ISC proliferation and regeneration (Park et al. 2018). Notably, *O-*GlcNAcylation directly modifies several DDR components, suggesting a potential crosstalk between metabolic regulation and genome maintenance (Konzman et al. 2020). Despite the independent roles of *O-*GlcNAcylation and steroid hormones in ISC regulation, how these signals converge and affect DNA integrity and stem cell behavior under stress or aging remains unclear. In particular, the interaction between *O-*GlcNAc and steroid hormone signaling molecules, such as ecdysone, has not been fully elucidated despite their acknowledgment as regulators of glucose metabolism and cell differentiation.

Adult stem cells are essential for maintaining tissue function and supporting development by acting as reservoirs for homeostasis and regeneration (Mannino et al. 2022). In particular, intestinal homeostasis depends on the precise balance between ISC proliferation and differentiation (Santos et al. 2018). This balance is tightly regulated by hormones, which exert their effects both inside and outside the intestinal environment (Grmai et al. 2024). Hormones are critical for developmental processes, including tissue growth, and they play diverse physiological roles in nutrient regulation and stress responses (Tavares et al. 2022). In addition, steroid hormones regulate intestinal metabolic pathways, thereby influencing ISC activity and overall gut function (Kandelouei et al. 2024). The *Drosophila* intestine serves as a powerful model system for studying adult stem cell biology, with implications for age-related and stem cell-derived diseases, such as cancer (Sanada et al. 2018; Mirzoyan et al. 2019; Funk et al. 2020; Rodriguez-Fernandez et al. 2020). It offers significant parallels to mammalian systems in terms of development, cellular composition, and genetic control (Kimble and Nusslein-Volhard 2022). The adult midgut epithelium consists of 4 distinct cell types: ISCs, undifferentiated progenitor cells known as enteroblasts (EBs), and differentiated cells, including absorptive enterocytes and secretory enteroendocrine cells (Micchelli and Perrimon 2006; Ohlstein and Spradling 2006). Our research demonstrated that *O-*GlcNAcylation is a crucial regulator of ISC and progenitor cell homeostasis in *Drosophila* (Na et al. 2020). These findings highlight the importance of *O-*GlcNAc in stem cell maintenance, functionality, and tissue development, particularly through the regulation of DDR and stress-induced proliferation (Na et al. 2020). In recent years, the global incidence of metabolic disorders has increased dramatically owing to an increase in obesity and aging populations (Saklayen 2018). These metabolic dysfunctions are intimately linked to age-associated diseases, such as cancer and type 2 diabetes (Sousa et al. 2022). Therefore, elucidating the molecular interplay between nutrient-sensitive pathways, such as *O-*GlcNAcylation, and hormonal cues, such as ecdysone signaling, is essential for understanding ISC dysregulation and tissue aging. This study aimed to uncover the functional crosstalk between ecdysone signaling and *O-*GlcNAcylation in the *Drosophila* adult midgut and investigate their combined impact on ISC proliferation and genomic stability under stress and aging conditions.

Although the individual roles of EcR and *O-*GlcNAcylation in ISC/EB regulation have been reported, their functional interactions remain largely unexplored. Ecdysone is a systemic hormonal signal, whereas *O-*GlcNAcylation serves as a cell-intrinsic nutrient sensor. Therefore, uncovering how these 2 evolutionarily conserved pathways intersect may reveal novel mechanisms, through which hormonal and metabolic cues are integrated to maintain ISC homeostasis and genomic integrity. This interaction is particularly relevant in the context of aging and stress, where both pathways are dysregulated and contribute to epithelial dysfunction. In this study, we aimed to investigate the functional crosstalk between EcR and *O-*GlcNAcylation and its impact on ISC proliferation and the DDR using the *Drosophila* adult midgut as a model.

## Materials and methods

### *Drosophila* stocks, culture, and husbandry

Fly stocks were maintained at 25 °C on standard food under a 12 h/12 h light/dark cycle. Food consisted of 15.8 g yeast, 9 g soy flour, 5.2 g agar, 67 g cornmeal, and 0.5% propionic acid. To avoid larval overpopulation, <30 adult flies per vial were transferred to new food vials every 2 to 3 d. The following stocks were used in this study: esg-Gal4, tub-Gal80ts, UAS-GFP/CyO [esg > GFP (gift from Bruce Edgar)]; Oregon-R (BDRC, #5); w1118 (BDRC, #3605); UAS-OGARNAi (BDRC, #82451); UAS-OGARNAi (VDRC, #106670); UAS-OGTRNAi (BDRC, #50909); UAS-OGTRNAi (VDRC, #18610); UAS-EcR (BDSC, #9450); and UAS-EcRRNAi (BDRC, #9327).

The GAL80ts system was used to temporally control transgene expression. Crosses were established and maintained at 22 °C to suppress GAL4 activity during development. After eclosion, adult female flies were collected within 6 to 12 h, sorted under brief CO_2_ anesthesia, and aged at 29 °C for 7 d depending on the experiment to activate transgene expression. Unless otherwise noted, all experiments were performed using adult female flies, as they showed more consistent intestinal morphology and ISC activity than males.

### Thiamet G feeding assay

Two-day-old adult flies were treated with 300 µM Thiamet G (Selleckchem) in standard media. Flies were maintained at 29 °C for 7 d, after which female midguts were dissected for analysis.

### 20HE feeding assay

Two-day-old flies were treated with 5 mM 20HE (Sigma-Aldrich) in standard media for 24 h at 29 °C. After feeding, the midguts were dissected and analyzed.

### OSMI feeding assay

Two-day-old adult flies were treated with 50 µM OSMI (Selleckchem) in standard media. Flies were maintained at 29 °C for 7 d, after which female midguts were dissected for analysis.

### Immunochemistry

Intact adult guts were dissected, fixed at room temperature for 1 h in 4% paraformaldehyde, washed with 0.1% Tween 20 in phosphate-buffered saline (PBST), and incubated overnight with primary antibodies at 4 °C. The primary antibodies used in this study include rabbit phospho-histone H3 (Ser10) (Millipore, Cat# 06–570, 1:500 dilution); mouse anti-green fluorescent protein (GFP) [Developmental Studies Hybridoma Bank (DSHB), Cat# DSHB-GFP-4C9, 1:100 dilution]; rabbit anti-GFP (Thermo Fisher Scientific, Cat# A11122, 1:500 dilution); mouse anti-*O-*GlcNAc (HGAC85) (Thermo Fisher Scientific, Cat# MA1-076, 1:50 dilution); rabbit phospho-ATM/ATR substrate motif [(pS/pT) QG] (Cell Signaling Technology, Cat# 6966S, 1:100 dilution); rabbit polyclonal anti-Histone H2AvD (pS137) (Rockland Immunochemicals, Cat# 600-401-914, 1:100 dilution); and mouse anti-ecdysone receptor (EcR-A) (Development Hydros Bank *Drosophila*, Cat# 15G1a(EcR-A), 1:100 dilution). The samples were then incubated for 2 h with secondary antibodies at 25 °C. The secondary antibodies used in this study included goat anti-mouse antibody Alexa Fluor 568, goat anti-mouse antibody Alexa Fluor 488, goat anti-rabbit antibody Alexa Fluor 568, and Goat anti-rabbit antibody Alexa Fluor 488 (Thermo Fisher Scientific, 1:300 dilution). After washing with PBST, slides were mounted using Vectashield. Images were acquired using a Zeiss LSM 700 system or an Olympus FY3000 confocal microscope with a 20× and 40× objective lens. All images were captured using identical laser power, gain, and exposure settings for each experimental group to allow accurate comparison. Image processing was performed using the Fiji software (ImageJ).

### Quantification of PH3-positive cells

The number of PH3-positive cells in the whole gut was counted for quantitative analysis. *N* represents the number of guts.

### Measurement of *O-*GlcNAc, EcR, pS/Tq, and γH2AVD fluorescence in ISCs/EBs

The fluorescence images of *O-*GlcNAc, EcR, pS/Tq, and γH2AVD staining were captured at the same exposure time in each experiment and were measured by quantifying the level of fluorescence (IHC staining) in individual ISCs normalized to the nearby background in Fiji software (ImageJ). The mean fluorescence was analyzed after excluding the mean of the background region (from 2 spots, excluding the nuclear portion in the posterior midgut), with the background fluorescence set to 0. At least 10 ISCs were quantified in each image, and >10 images (>1 image per fly) were used to calculate the average fluorescence intensity of the ISCs in each fly; *n* represents the number of cells.

### Statistical analysis

Data representation and statistical analyses were performed using GraphPad Prism software. Statistical analysis was performed using a *t*-test, and multiple comparisons were performed using one-way ANOVA. All experiments were performed with at least 3 independent biological replicates, each lasting approximately 15 min. *N* represents the number of guts, and *n* represents the number of cells.

## Results

### EcR expression is related to ISC proliferation

**Fig. 1.**
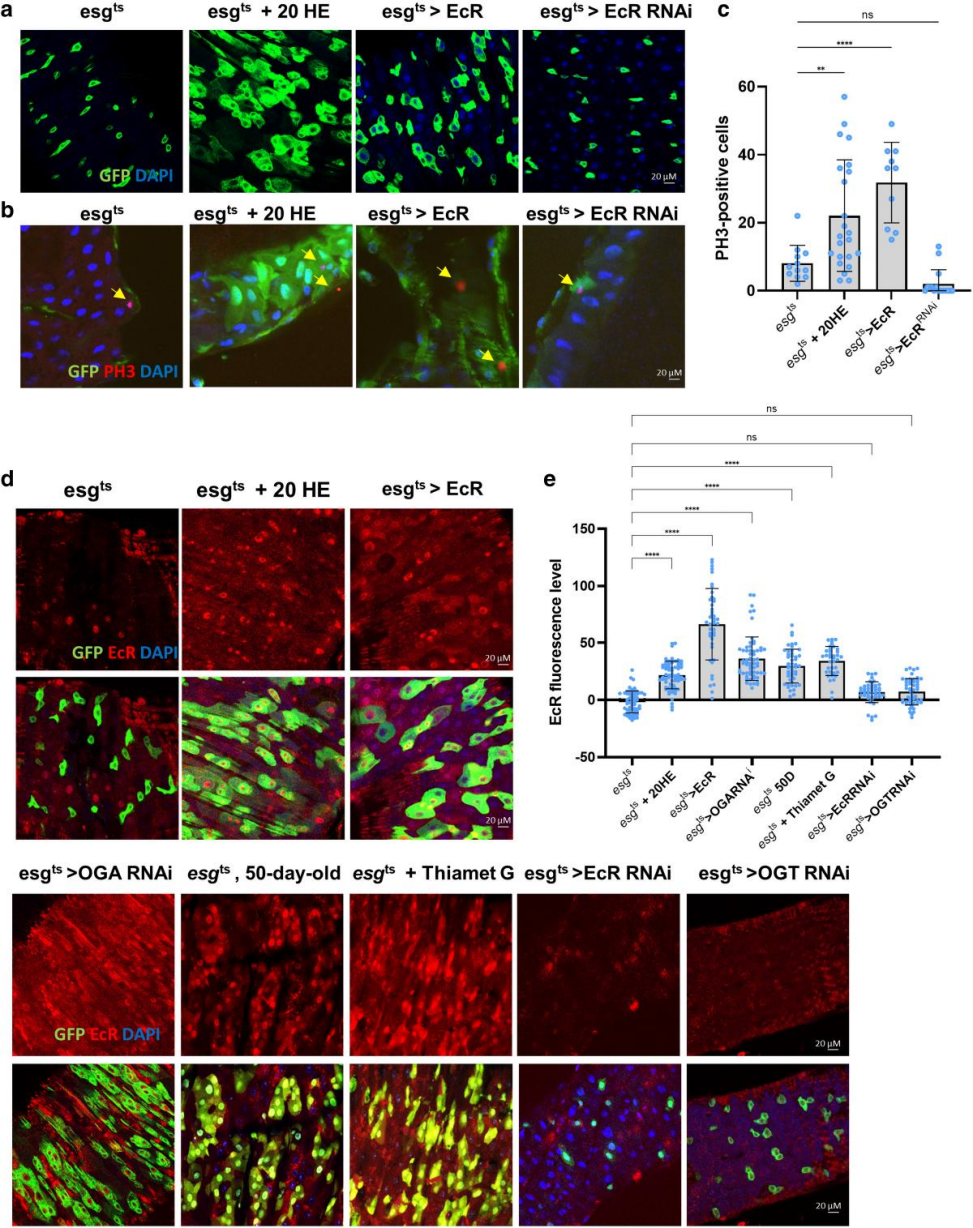
EcR expression is related to ISC proliferation. a) Immunofluorescence staining to analyze esg-GFP (green) in the fly midgut. b) Immunofluorescence staining to analyze phospho-H3 (PH3, red) in the fly midgut. c) Quantification of PH3-positive cells (mitotic marker) in fly midguts (*N*; *esg^ts^* = 12, *esg^ts^* + 20HE = 23, *esg^ts^* > EcR = 10, and *esg^ts^* > EcR^RNAi^ = 16). d) Immunofluorescence staining of EcR (red) in esg-GFP-positive cells (green) in the fly midgut. e) Quantification of EcR mean fluorescence per esg-GFP-positive cell (*n*; *esg^ts^* = 63, *esg^ts^* + 20HE = 70, *esg^ts^* > EcR = 43, *esg^ts^* > OGA^RNAi^ = 61, 50-day-old *esg^ts^* = 49, *esg^ts^* + 20HE = 37, *esg^ts^* > EcR^RNAi^ = 45, and *esg^ts^* > OGT^RNAi^ = 50). *N* represents the number of guts. *n* represents the number of cells. Data are represented as means ± SD. Yellow arrows indicate PH3-positive cells. ****P < 0.0001, ***P < 0.001, ^**^P < 0.01, ^*^P < 0.05.

In the *Drosophila* intestine, ISCs proliferate more extensively in females than in males, contributing to age-related phenotypes, such as dysplasia and intestinal tumors (Ahmed et al. 2020). Previous reports have shown that 20HE promotes ISC proliferation and increases gut size in female *Drosophila* (Ahmed et al. 2020). Na et al. (2020) further demonstrated that an age-dependent increase in *O-*GlcNAc levels correlated with ISC proliferation. In the present study, we investigated the relationship between EcR expression and *O-*GlcNAcylation in the maintenance of intestinal homeostasis (Na et al. 2020). To determine the relationship between EcR expression and ISC proliferation, we used EcR overexpression (UAS-EcR), EcR knockdown (UAS-EcRRNAi), and 20HE treatment using esg-GAL4, UAS-GFP, Tub-GAL80TS flies (ISC/EB cell-specific inducible GAL4, *esg^t^*^s^) (Micchelli and Perrimon 2006; Ohlstein and Spradling 2006). After incubation at 29 °C for 7 d, we observed a significant increase in the number of esg-GFP-positive cells and PH3-positive cells (mitotic cells) in *esg^ts^* > EcR- and 20HE-treated flies compared to controls (Fig. 1a to c). Conversely, these numbers did not increase in *esg^t^*^s^ > EcR^RNAi^ flies (Fig. 1a to c). Anti-EcR staining was performed to assess EcR expression. EcR expression increased in *esg^t^*^s^ > EcR- and 20HE-treated flies compared to that in controls (Fig. 1d,e). Interestingly, EcR expression was also upregulated in 50-day-old *esg^ts^* flies, *esg^t^*^s^ > OGA^RNAi^ flies (which block *O-*GlcNAc removal), and flies treated with Thiamet G (which increases *O-*GlcNAcylation) (Fig. 1d,e). Conversely, EcR expression did not increase in *esg^t^*^s^ > EcR^RNAi^ or *esg^t^*^s^ > OGT^RNAi^ flies (which reduce *O-*GlcNAcylation) (Fig. 1d,e). These results suggest that EcR expression increases with aging, EcR overexpression, and increased *O-*GlcNAcylation and is associated with increased ISC proliferation. These results suggest an association with age-related dysplasia.

### EcR expression elevated *O-*GlcNAc levels and DDR in ISC/EB

**Fig. 2.**
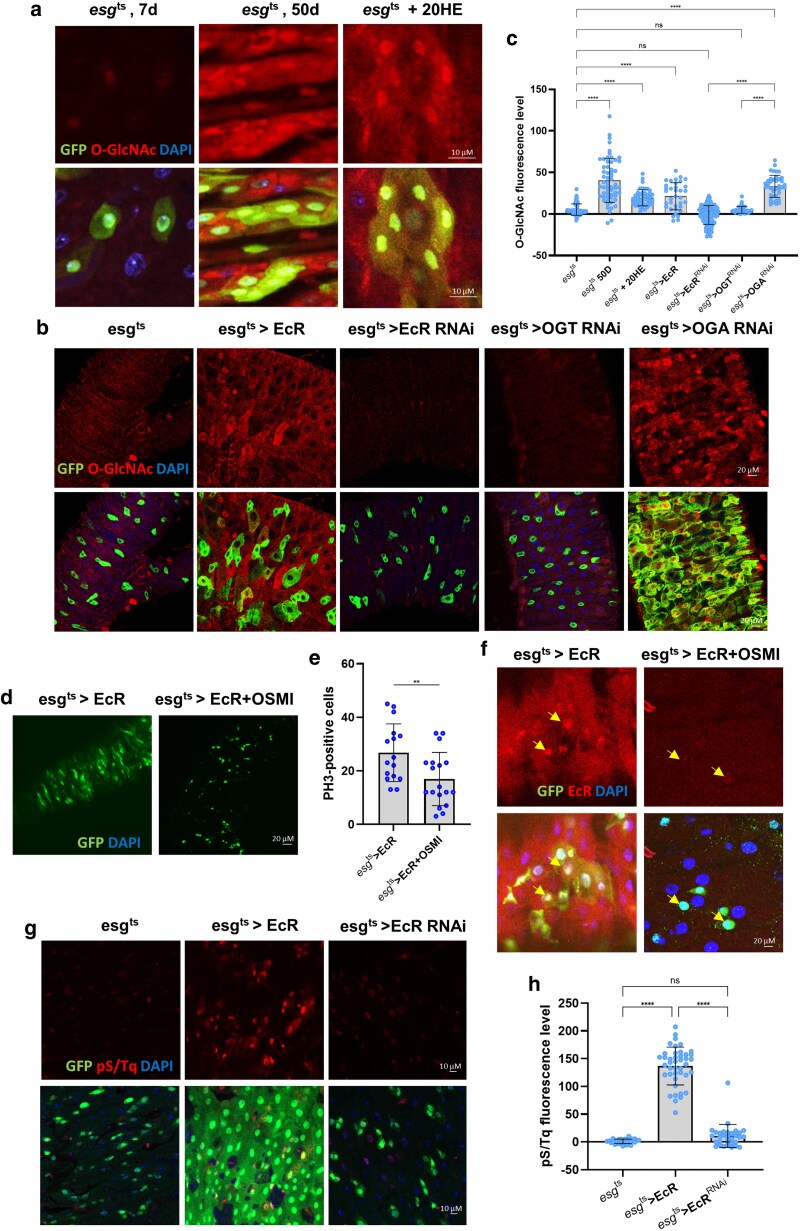
EcR expression elevated *O-*GlcNAc levels and ATM/ATR activity in ISC/EB. a) Immunofluorescence staining of *O-*GlcNAc (red) in esg-GFP-positive cells (green) from 7-day-old, 50-day-old, and 5 mM 20HE-treated fly midguts. b) Immunofluorescence staining of *O-*GlcNAc (red) in esg-GFP-positive cells (green) from *esg^ts^*, *esg^t^*^s^ > EcR, *esg^t^*^s^ > EcR^RNAi^, and *esg^t^*^s^ > OGA^RNAi^ fly midguts. c) Quantification of *O-*GlcNAc mean fluorescence per esg-GFP-positive cell. (*n*; *esg^ts^* = 66, 50-day-old *esg^ts^* = 59, *esg^ts^* + 20HE = 46, *esg^ts^* > EcR = 35, *esg^ts^* > EcR^RNAi^ = 91, *esg^ts^* > OGT^RNAi^ = 39, and *esg^ts^* > OGA^RNAi^ = 40). d) Immunofluorescence staining of GFP (green) in *esg^t^*^s^ > EcR and *esg^t^*^s^ > EcR + OSMI fly midguts. e) Quantification of PH3-positive cells (mitotic marker) in fly midguts (*N*; *esg^t^*^s^ > EcR = 16 and *esg^t^*^s^ > EcR + OSMI = 18). f) Immunofluorescence staining of EcR (red) in esg-GFP-positive cells (green) in *esg^t^*^s^ > EcR and *esg^t^*^s^ > EcR + OSMI fly midguts. g) Immunofluorescence staining of pS/Tq (red) in esg-GFP-positive cells (green) from *esg^t^*^s^, *esg^t^*^s^ > EcR, and *esg^t^*^s^ > EcR^RNAi^ fly midguts. h) Quantification of pS/Tq mean fluorescence per esg-GFP-positive cell (*n*; *esg^ts^* = 29, *esg^t^*^s^ > EcR = 42, and *esg^t^*^s^ > EcR^RNAi^ = 31); *n* represents the number of cells. Yellow arrows indicate GFP-positive cells. Data are presented as means ± SD. ****P < 0.0001, ***P < 0.001, ^**^P < 0.01, ^*^P < 0.05.

As shown in Fig. 1, EcR expression was upregulated in *esg^t^*^s^ > OGA^RNAi^- and Thiamet G-treated flies, suggesting a possible contribution to elevated *O-*GlcNAc levels. Previous reports have indicated that *O-*GlcNAc levels are elevated under *esg^ts^* > OGA^RNAi^, aging, and high-sucrose conditions. In contrast, *O-*GlcNAc levels did not increase in *esg^ts^* > OGT^RNAi^ midguts (Na et al. 2020). To further explore the relationship between EcR and *O-*GlcNAcylation, we examined *O-*GlcNAc expression in ISCs/EBs following EcR overexpression, knockdown, and 20HE treatment. Consistent with previous findings, *O-*GlcNAc levels increased in 50-day-old flies and OGA-knockdown midguts (Fig. 2a to c) (Na et al. 2020). Similarly, EcR overexpression and 20HE treatment elevated *O-*GlcNAc levels in ISCs/EBs (Fig. 2a to c). Conversely, *O-*GlcNAc levels were reduced in *esg^t^*^s^ > EcR^RNAi^ or *esg^t^*^s^ > OGT^RNAi^ flies compared to those in controls (Fig. 2a to c). We investigated whether blocking O-GlcNAc accumulation affected ISC proliferation under EcR overexpression. We treated flies overexpressing EcR with OSMI, a selective OGT inhibitor. The results showed that OSMI treatment attenuated both elevated ISC proliferation and increased EcR expression induced by EcR overexpression, suggesting that reduced *O*-GlcNAcylation impairs EcR-mediated ISC proliferation at multiple levels (Fig. 2d to f). These results indicate that EcR activity promotes *O-*GlcNAc accumulation, which in turn supports ISC proliferation.

Our data and those of previous studies show that EcR expression is correlated with ISC proliferation (Fig. 1) (Ahmed et al. 2020). Given that ISC hyperproliferation is linked to DNA damage accumulation (Park et al. 2012) and that *O-*GlcNAcylation regulates DDR-related genes through feedback mechanisms, we hypothesized that EcR may influence genome integrity via *O-*GlcNAc signaling. Indeed, γH2AVD and ATM/ATR (pS/TQ) levels were markedly elevated in *esg^ts^* > OGA^RNAi^ midguts, as well as under aging and high-sucrose conditions, whereas *esg^ts^* > OGT^RNAi^ midguts showed only partial γH2AVD induction and exhibited impaired DNA damage protection due to reduced *O-*GlcNAcylation (Na et al. 2020) (Supplementary Fig. 1). Because ISCs are highly sensitive to genomic instability, pS/TQ serves as a sensitive readout for DDR activation in these cells (Park et al. 2018). To test whether EcR activity contributes to DNA damage accumulation in ISCs, we examined the phosphorylation of pS/TQ motifs that mark the early activation of DDR following double-strand breaks or replication stress. Our results showed that EcR overexpression increased pS/TQ levels in ISCs/EBs, whereas EcR knockdown abrogated pS/TQ induction (Fig. 2g,h). These findings suggest that EcR promotes DNA damage accumulation in ISCs/EBs, potentially through *O-*GlcNAc, the O-GlcNAc-mediated modulation of DDR pathways.

### EcR is required for ISC proliferation and DNA damage accumulation during 20HE treatment in ISC/EB

**Fig. 3.**
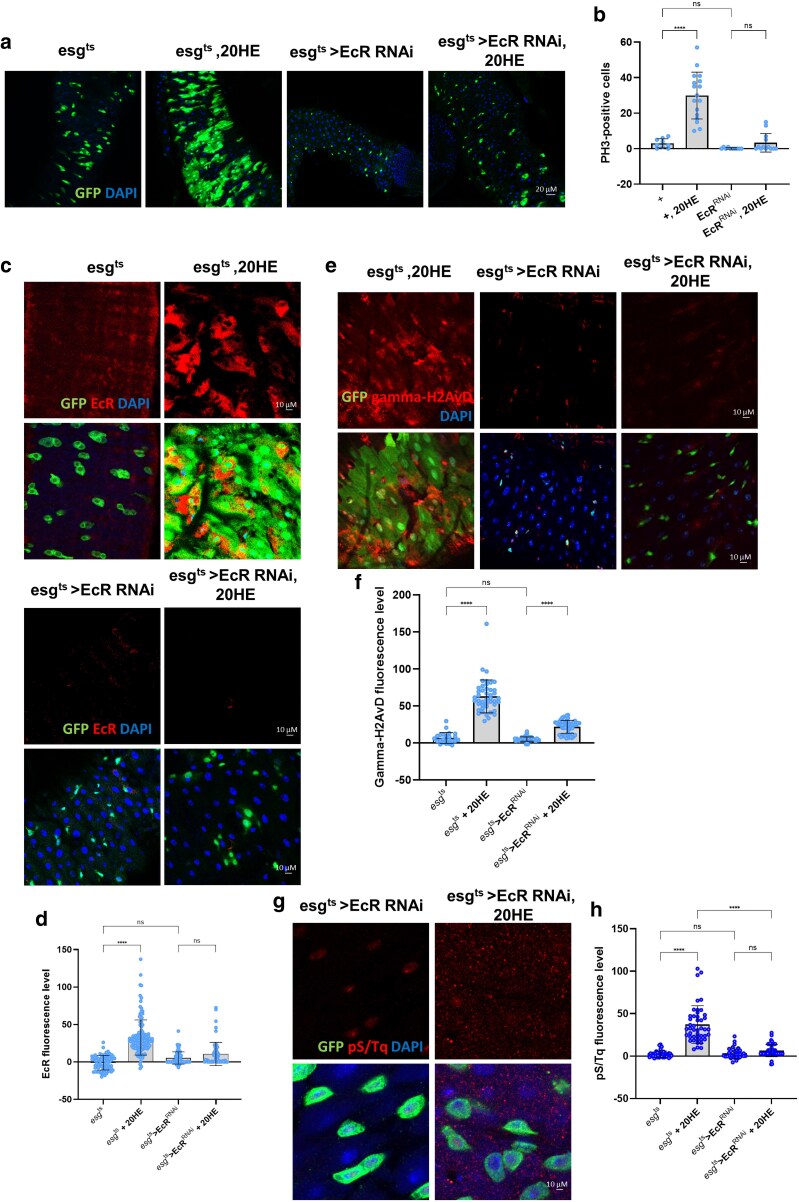
EcR is required for ISC proliferation and DNA damage accumulation during 20HE treatment in ISCs/EBs. a) Immunofluorescence staining to analyze esg-GFP (green) in *esg^t^*^s^, *esg^ts^* + 20HE, *esg^t^*^s^ > EcR^RNAi^, and *esg^t^*^s^ > EcR^RNAi^ + 20HE fly midguts. b) Quantification of PH3-positive cells (mitotic marker) in fly midguts (*N*; *esg^ts^* = 17, *esg^ts^* + 20HE = 21, *esg^t^*^s^ > EcR^RNAi^ = 20, and *esg^t^*^s^ > EcR^RNAi^ + 20HE = 11). c) Immunofluorescence staining of EcR (red) in esg-GFP-positive cells (green) from *esg^t^*^s^, *esg^ts^* + 20HE, *esg^t^*^s^ > EcR^RNAi^, and *esg^t^*^s^ > EcR^RNAi^ + 20HE fly midguts. d) Quantification of EcR mean fluorescence per esg-GFP-positive cell (*n*; *esg^ts^* = 77, *esg^ts^* + 20HE = 123, *esg^t^*^s^ > EcR^RNAi^ = 77, and *esg^t^*^s^ > EcR^RNAi^ + 20HE = 67). e) Immunofluorescence staining of γH2AVD (red) in esg-GFP-positive cells (green) from *esg^ts^* + 20HE, *esg^ts^* > EcR^RNAi^, and *esg^ts^* > EcR^RNAi^ + 20HE fly midguts. f) Quantification of γH2AVD mean fluorescence per esg-GFP-positive cell. (*n*; *esg^ts^* = 24, *esg^ts^* + 20HE = 44, *esg^t^*^s^ > EcR^RNAi^ = 46, and *esg^t^*^s^ > EcR^RNAi^ + 20HE = 52). g) Immunofluorescence staining of pS/Tq (red) in esg-GFP-positive cells (green) from *esg^t^*^s^ > EcR^RNAi^ and *esg^t^*^s^ > EcR^RNAi^ + 20HE fly midguts. h) Quantification of pS/Tq mean fluorescence per esg-GFP-positive cell (*n*; *esg^ts^* = 51, *esg^ts^* + 20HE = 45, *esg^ts^* > EcR^RNAi^ = 33, and *esg^t^*^s^ > EcR^RNAi^ + 20HE = 50). *N* represents the number of guts; *n* represents the number of cells. Data are presented as means ± SD^. ****^P < 0.0001, ***P < 0.001, ^**^P < 0.01, ^*^P < 0.05.

Our data indicated that EcR expression induces ISC proliferation and DNA damage accumulation in ISCs/EBs (Fig. 2). Next, we investigated whether EcR was required for DDR and proliferation in ISCs/EBs under 20HE treatment. We examined whether EcR knockdown prevented 20HE-induced hyperproliferation and found a significant lack of increase in esg-positive cells and PH3-positive ISCs/EBs in 20HE-treated *esg^t^*^s^ > EcR^RNAi^ midguts compared to 20HE-treated *esg^t^*^s^ midguts (Fig. 3a,b). Furthermore, EcR expression did not change in esg-positive cells of 20HE-treated *esg^t^*^s^ > EcR^RNAi^ midguts compared to that in 20HE-treated controls (Fig. 3c,d). Indeed, 20HE treatment led to either partial induction or no significant change in γH2AVD levels in esg-positive cells of the *esg^t^*^s^ > EcR^RNAi^ midgut compared to 20HE-treated *esg^ts^* control flies (Fig. 3e,f). Additionally, pS/TQ expression showed either partial induction or no significant change in the *esg^t^*^s^ > EcR^RNAi^ midgut with 20HE treatment compared to that in 20HE-treated *esg^ts^* control flies (Fig. 3g,h). Strikingly, loss of EcR resulted in a significant decrease in the DDR markers γH2AVD and pS/TQ (Fig. 3e to h) and EcR in 20HE-treated cells compared to 20HE-treated control esg-positive cells. These results indicate that EcR is essential for 20HE-induced ISC proliferation and accumulation of DNA damage.

### EcR is required for 20HE-induced *O-*GlcNAc expression and stem cell proliferation

**Fig. 4.**
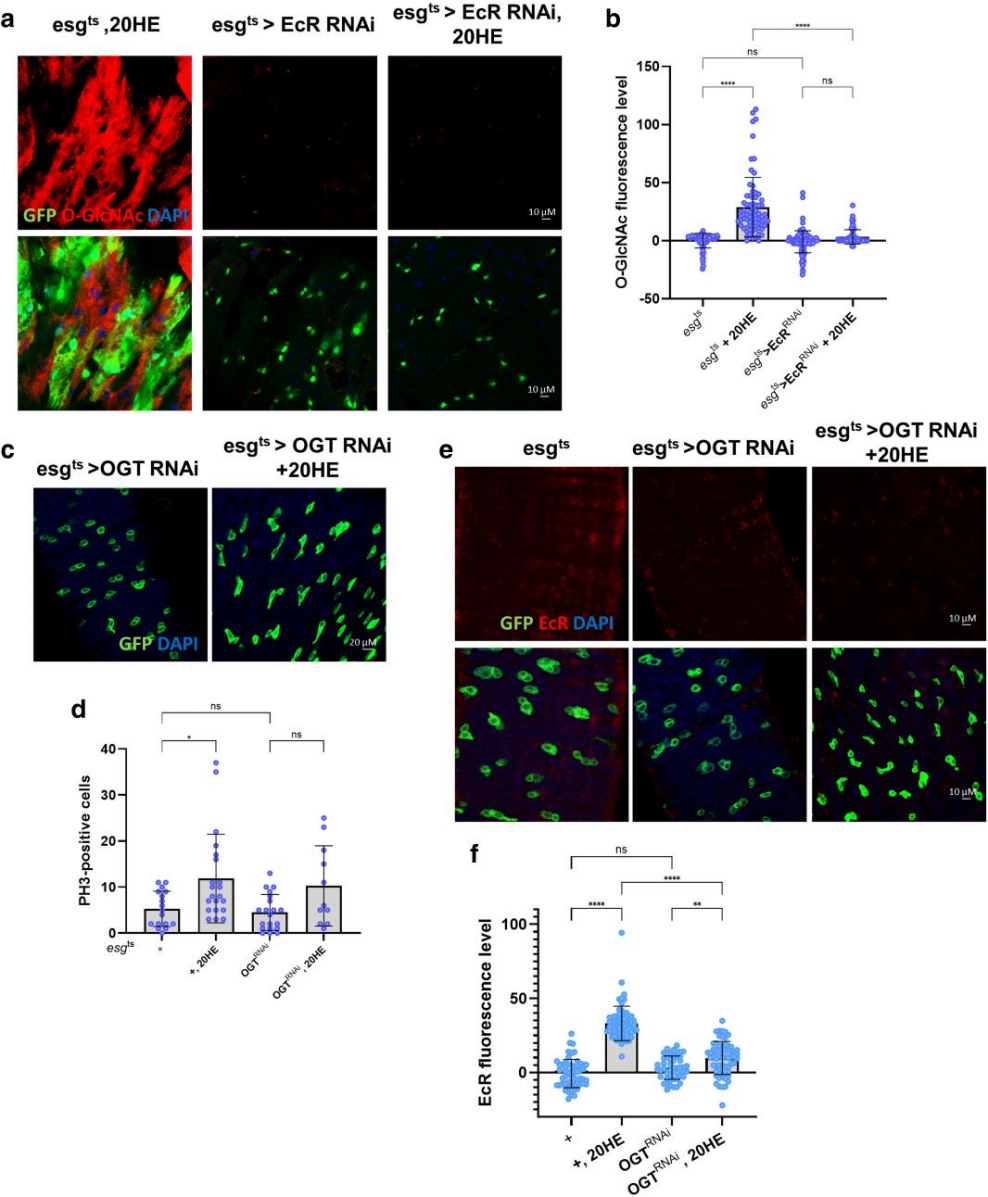
EcR is required for 20HE-induced *O-*GlcNAc expression and stem cell proliferation. a) Immunofluorescence staining of *O-*GlcNAc (red) in esg-GFP-positive cells (green) from *esg^ts^* + 20HE, *esg^ts^* > EcR^RNAi^, and *esg^t^*^s^ > EcR^RNAi^ + 20HE fly midguts. b) Quantification of *O-*GlcNAc mean fluorescence per esg-GFP-positive cell (*n*; *esg^ts^* = 81, *esg^ts^* + 20HE = 73, *esg^t^*^s^ > EcR^RNAi^ = 123, and *esg^t^*^s^ > EcR^RNAi^ + 20HE = 79). c) Immunofluorescence staining to analyze esg-GFP (green) in fly midguts. d) Quantification of PH3-positive cells (mitotic marker) in fly midguts. (*N*; *esg^ts^* = 17, *esg^ts^* + 20HE = 21, *esg^ts^* > OGT^RNAi^ = 20, and *esg^t^*^s^ > OGT^RNAi^ + 20HE = 11). e) Immunofluorescence staining of EcR (red) in esg-GFP-positive cells (green) in 7-day-old *esg^t^*^s^ and *esg^t^*^s^ > OGT^RNAi^ fly midguts with or without 5 mM 20HE treatment. f) Quantification of *O-*GlcNAc mean fluorescence per esg-GFP-positive cell (*n*; *esg^ts^* = 56, *esg^ts^* + 20HE = 65, *esg^ts^* > OGT^RNAi^ = 50, and *esg^t^*^s^ > OGT^RNAi^ + 20HE = 64). *N* represents the number of guts; *n* represents the number of cells. Data are presented as means ± SD. ****P < 0.0001, ***P < 0.001, ^**^P < 0.01, ^*^P < 0.05.

As shown in Figs. 1 and 2, EcR expression was associated with *O-*GlcNAc expression. To further clarify the response of ISCs and EBs to 20HE treatment, we examined changes in *O-*GlcNAc levels following EcR knockdown, as well as OGT knockdown under 20HE treatment. *O-*GlcNAc levels were upregulated in ISCs/EBs following 20HE treatment. However, EcR knockdown abolished *O-*GlcNAc expression regardless of 20HE treatment (Fig. 4a,b). Next, we sought to determine whether OGT knockdown cells responded to 20HE treatment. As expected, the number of PH3-positive cells in the midgut increased as a result. However, in *esg^ts^* > OGT^RNAi^ flies, neither PH3-positive nor esg-GFP-positive cells increased following 20HE treatment compared to the untreated controls (Fig. 4c,d). Furthermore, EcR levels were not elevated in *esg^ts^* > OGT^RNAi^ flies treated with 20HE compared to the controls (Fig. 4e,f). Together, these results confirm that EcR is essential for 20HE-induced *O-*GlcNAcylation and ISC proliferation. Both EcR and OGT are required for the upregulation of *O-*GlcNAc levels and mitotic activity in response to 20HE stimulation.

## Discussion

In this study, we investigated the interplay between steroid hormone signaling and nutrient-sensitive *O-*GlcNAcylation in the regulation of ISC activity and homeostasis in the *Drosophila* midgut. Our results revealed a functional link between EcR and *O-*GlcNAc modification, which may have important implications for ISC proliferation, DNA damage accumulation, and metabolic regulation in the context of aging and disease.

Ecdysone binds to specific nuclear receptors and regulates gene expression, significantly influencing stem cell differentiation and proliferation (Zipper et al. 2020). Several studies on insects have shown that ecdysone promotes both the self-renewal and differentiation of stem cells, thereby inducing proliferation (Ahmed et al. 2020; Zipper et al. 2020; Karanja et al. 2022). The effects of ecdysone on stem cells vary depending on the cellular context and environmental stimuli. In some cases, ecdysone enhances stem cell activation when acting in concert with external cues (Ables and Drummond-Barbosa 2010; Homem et al. 2014). Thus, ecdysone is a key hormone regulating developmental and physiological processes in insects (Yamanaka et al. 2013). We first confirmed that EcR expression was correlated with ISC proliferation, consistent with previous findings (Ahmed et al. 2020), and demonstrated that both EcR overexpression and exogenous 20HE treatment led to an increased number of proliferative ISCs/EBs (Fig. 1). Conversely, EcR knockdown suppressed ISC proliferation even in the presence of 20HE, confirming that EcR is a critical driver of intestinal epithelial proliferation (Fig. 1). Interestingly, we also observed that EcR expression was elevated in aged midguts, as well as under conditions of elevated *O*-GlcNAcylation, such as in OGA-knockdown flies or after Thiamet G treatment (Fig. 2). This observation suggests a regulatory interaction between EcR and *O-*GlcNAc signaling. Our analysis showed that both EcR expression and 20HE treatment induced *O-*GlcNAc accumulation in ISCs/EBs, whereas EcR or OGT knockdown abrogated this effect (Fig. 4). These findings suggest a positive feedback loop, where EcR activity depends on and promotes *O-*GlcNAcylation, integrating the hormonal and metabolic controls of stem cell behavior.

*O-*GlcNAc transferase and *O-*GlcNAcase regulate the dynamic cycling of *O-*GlcNAcylation in a nutrient- and stress-dependent manner (Konzman et al. 2020). *O-*GlcNAcylation contributes to tissue development and has been implicated in cancer malignancy (Ferrer et al. 2016). It also modulates cellular stress responses, including DNA damage, by influencing key DNA repair kinases, such as ATM. DNA damage and oxidative stress lead to increased *O-*GlcNAc levels, and *O-*GlcNAc has been shown to promote ATM activation in response to double-strand breaks (Konzman et al. 2020; Na et al. 2020). Given that ecdysone signaling can regulate genes involved in cell cycle progression, apoptosis, and stress resistance (Guo et al. 2016), it is plausible that the ecdysone and *O-*GlcNAc pathways converge to modulate DDR.

Importantly, we found that EcR overexpression led to increased DNA damage accumulation in ISCs/EBs, as indicated by the elevated levels of γH2AVD and pS/TQ, well-established markers of DNA double-strand breaks, and activation of the DDR pathway (Fig. 2). Because proliferative stem cells are highly vulnerable to genotoxic stress, our data support a model where excessive or prolonged EcR activity may compromise genomic integrity and potentially contribute to age-related dysplasia and epithelial dysfunction. Furthermore, we demonstrated that EcR is required for ISC proliferation, as well as DNA damage accumulation in response to 20HE treatment (Fig. 3). EcR or OGT knockdown blocked 20HE-induced ISC expansion and prevented the upregulation of DDR markers, indicating that both EcR and *O-*GlcNAcylation are essential mediators of steroid hormone-driven ISC activation (Fig. 4). Reactive oxygen species (ROS) are critical factors that link DNA damage and stem cell stress. ROS can cause DNA damage, and DNA damage can in turn increase ROS production, forming a detrimental feedback loop (Srinivas et al. 2019). Ecdysone induces ROS production in *Drosophila*, and ROS, in turn, modulate signaling pathways, including those involving EcR (Kannangara et al. 2021). During development and metamorphosis, ecdysone-induced ROS regulate cell growth, differentiation, and programed cell death, helping to eliminate unnecessary tissues (Toshniwal et al. 2019). Thus, ROS may amplify ecdysone-driven ISC proliferation, particularly during DNA damage and stress.

These findings expand on previous studies linking *O-*GlcNAcylation to DNA repair by placing EcR at the intersection of hormonal signaling and nutrient sensing during adult stem cell regulation. Taken together, while EcR promotes ISC proliferation and may contribute to age-associated epithelial phenotypes, its interaction with *O-*GlcNAcylation suggests a metabolic gatekeeper role that integrates hormonal and nutritional signals to balance regenerative activity and genome integrity. As the prevalence of metabolic and age-related diseases, including cancer and diabetes, continues to increase, understanding how steroid hormones and nutrient signaling converge at the adult stem cell level may offer promising new therapeutic avenues.

## Supplementary Material

jkaf190_Supplementary_Data

## Data Availability

The authors affirm that all the data necessary to confirm the conclusions of the article are presented in the article and figures. All the data are available in the published article. Supplemental material available at G3 online.
