# Supplementary material for: Integrated ecdysone and O-linked N-acetylglucosamine signaling coordinates intestinal stem cell proliferation in Drosophila midgut
Source: G3 (Bethesda). 2025 Aug 19;15(11):jkaf190. doi: 10.1093/g3journal/jkaf190 (PMC12611247; doi:10.1093/g3journal/jkaf190)
Supplement: jkaf190_Supplementary_Data [file jkaf190_supplementary_data.zip › Supplemental_Figure_Legend_G3-2025-406052.docx]

**Supplemental Figure Legend**

**Supplemental Figure 1. γH2AVD Expression in OGT- and OGA-Knockdown ISCs/EBs.**

Immunofluorescence staining of γH2AVD (red) in esg-GFP-positive cells (green) from *esg*^ts^, *esg^t^*^s^>OGA^RNAi^, and *esg^t^*^s^>OGT^RNAi^ fly midguts.
