# Supplementary figures and images for: Integrated ecdysone and O-linked N-acetylglucosamine signaling coordinates intestinal stem cell proliferation in Drosophila midgut
Source: G3 (Bethesda). 2025 Aug 19;15(11):jkaf190. doi: 10.1093/g3journal/jkaf190 (PMC12611247; doi:10.1093/g3journal/jkaf190)

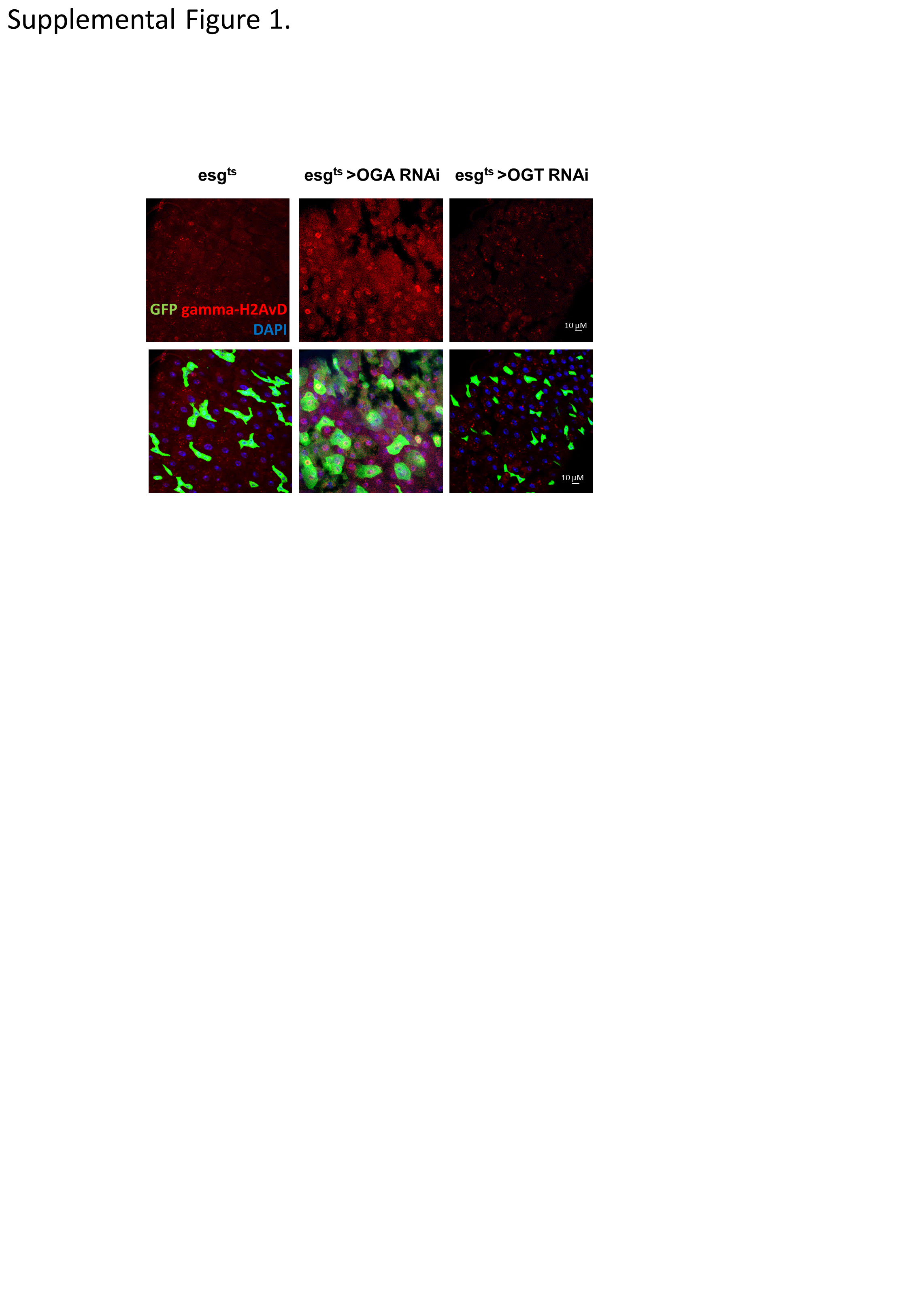

Supplement: jkaf190_Supplementary_Data [file jkaf190_supplementary_data.zip › Supplemental_Figure_1_G3-2025-406052.tif]
